# Intolerance of COVID-19-Related Uncertainty and Negative Emotions among Chinese Adolescents: A Moderated Mediation Model of Risk Perception, Social Exclusion and Perceived Efficacy

**DOI:** 10.3390/ijerph18062864

**Published:** 2021-03-11

**Authors:** Qi Li, Ronglei Luo, Xiaoya Zhang, Guangteng Meng, Bibing Dai, Xun Liu

**Affiliations:** 1 Beijing Key Laboratory of Learning and Cognition, Department of Psychology, Capital Normal University, Beijing 100048, China; liqi@psych.ac.cn; 2 Beijing Advanced Innovation Center for Imaging Technology, Capital Normal University, Beijing 100048, China; 3 Department of Psychiatry and Psychology, School of Basic Medical Sciences, Tianjin Medical University, Tianjin 300070, China; luoronglei@tmu.edu.cn; 4 CAS Key Laboratory of Behavioral Science, Institute of Psychology, Beijing 100101, China; zhangxiaoya@psych.ac.cn (X.Z.); menggt@psych.ac.cn (G.M.); liux@psych.ac.cn (X.L.); 5 Department of Psychology, University of Chinese Academy of Sciences, Beijing 100101, China

**Keywords:** COVID-19, intolerance of uncertainty, risk perception, social exclusion, perceived efficacy, negative emotions, adolescent

## Abstract

The uncertainty caused by the COVID-19 pandemic has exacerbated negative emotions, especially among adolescents, who feel unable to tolerate the uncertainty of the epidemic. However, the mechanism by which the intolerance of COVID-19-related uncertainty (COVID-19 IU) affects negative emotions in adolescents remains unclear. This study explored the underlying mechanism from COVID-19 IU to negative emotions using a moderated mediation model in adolescents. In total, 3037 teenagers completed a cross-sectional survey including measures of COVID-19 IU, risk perception, social exclusion, perceived efficacy, and negative emotions. The results showed that COVID-19 IU positively predicted negative emotions and that risk perception and social exclusion mediated this relationship. In addition, both the direct effect of COVID-19 IU on negative emotions and the mediating effect of risk perception on this relationship were moderated by perceived efficacy; in particular, COVID-19 IU had a greater impact on negative emotions among adolescents with lower levels of perceived efficacy. These findings suggest that COVID-19 IU is closely associated with negative emotions among adolescents and that effective measures should be taken to enable adolescents to improve their perceived efficacy and develop a reasonable perception of risk, help them eliminate the stigma of the disease, and strengthen their connections with society.

## 1. Introduction

The crisis facing the world in 2021 was the COVID-19 pandemic. Despite the various measures taken by governments to contain disease spread, the world remained in a state of pandemic, and the numbers of confirmed cases and deaths were out of control. This could be attributed to limited understanding of the disease and the lack of effective vaccines and treatments. By 6 March 2021, there were more than 115 million confirmed cases and over 2.5 million deaths [1]. As the number of global cases continued to increase, unpredictable repeat outbreaks occurred in some areas.

The extreme uncertainty brought about by the COVID-19 pandemic is causing anxiety, depression and other mental health problems [2,3,4,5]. In particular, adolescents with weak emotional regulation capacity are at a higher risk of developing multiple psychiatric disorders [6,7]. In addition to the threat of the pandemic, they are also experiencing prolonged school closure, home confinement and family conflicts, which can lead to serious emotional problems [5,8,9,10]. Therefore, it is of great significance to explore the mechanism by which negative emotions are generated in adolescents during the pandemic to help them regulate their emotions.

The integrative model of uncertainty tolerance suggests that the perception of uncertainty is associated with emotions directly or indirectly through cognitive appraisal, and in this process, individual factors, such as dispositional optimism and efficacy, have a moderating effect [11]. Based on this theory, this research proposes a moderated mediation model to explore the mechanism by which negative emotions are generated in adolescents during the COVID-19 crisis. In this model, intolerance of COVID-19-related uncertainty (COVID-19 IU) is a tendency to be unable to tolerate the uncertainty of COVID-19-related events, which directly influences one’s emotional state. In addition, COVID-19 IU, as a cognitive bias, also has an impact on emotions through the appraisal of threats. This appraisal involves two aspects. First, people evaluate the threats of disease to health, and those with higher COVID-19 IU are more likely to overestimate the risk of being infected. Second, the threats to interpersonal relationships are also evaluated. Individuals with higher COVID-19 IU tend to overestimate the social rejection and exclusion patients experience due to lockdown and social distancing. Additionally, as an individual trait, perceived efficacy, which involves cognitions related to the effectiveness of the recommended coping response and individuals’ ability to perform this response [12], may play an important role in moderating these processes. Therefore, this model is of great practical significance for understanding adolescents’ reactions to the uncertainty of the pandemic and also for helping them to regulate negative emotions.

### 1.1. Intolerance of COVID-19-Related Uncertainty and Negative Emotions

Intolerance of uncertainty (IU) describes an individual’s dispositional incapacity to endure an aversive response triggered by the perceived absence of salient, key, or sufficient information and is sustained by an associated perception of uncertainty [13]. Studies involving adults have found that IU is associated with a higher level of worry [14] and can predict anxiety [15]. Compared to general ambiguous life events, specific viral threats induce greater anxiety in individuals with high IU [16]. In the context of the COVID-19 pandemic, IU has also been shown to be an important predictor of fear. Moreover, IU is associated with adolescents’ worry and anxiety [17,18,19] as is the case in adults [20]. Nevertheless, as the COVID-19 pandemic is an event of great uncertainty, whether COVID-19 IU is closely related to adolescents’ negative emotions still requires further evidence.

### 1.2. Risk Perception and Social Exclusion as Mediators

Adolescents could become infected with and spread COVID-19, resulting in serious mental health problems (e.g., depression, anxiety, and post—traumatic stress disorder), the development of serious physical illness (e.g., multi-system inflammatory syndrome), and even death. During this pandemic, people experiencing IU perceive and appraise risks frequently, which may lead to negative emotions. Risk perception involves people’s subjective assessment of the probability of a specified type of accident occurring and how concerned they are with the consequences [21]. First, individuals with high COVID-19 IU are more likely to perceive a greater risk and overestimate their own probability of infection. It may be that individuals with high IU traits tend to make negative evaluations and overestimate risks [14]. In addition, high IU could lead to increased health monitoring [22]. Individuals with high IU may seek more threat-relevant information, which could increase their cognition of disease risk. Compared to adults, adolescents hold a less optimistic attitude toward avoiding injuries and illness [23]; that is, they perceive more risks. Second, higher risk perception induces more negative emotions. During the severe acute respiratory syndrome coronavirus outbreak, risk perception was positively correlated with fear and depression [24,25]. H1N1 research also shows that higher-IU individuals are prone to perceiving a pandemic as threatening, which, in turn, predicts higher anxiety [26]. Despite the established outcome, whether negative emotions are induced by risk assessment when adolescents are facing COVID-19 IU is still unknown.

Since COVID-19 is highly contagious, those infected are not only the victims of the pandemic but also vectors spreading the virus. Hence, social exclusion resulting from quarantine and lockdown may exacerbate their negative emotions. Social exclusion is usually defined as remaining separated from others [27]. During the COVID-19 pandemic, social exclusion mainly manifests in avoidance and rejection of both infected and potential patients. On the one hand, people adopt a series of avoidance behaviors to survive and stay away from individuals who carry the virus [28,29,30]. For instance, during the H1N1 pandemic, 76.5% of participants reported avoiding going out or visiting crowded places to lower their contact with infected persons to the utmost extent. Higher-IU individuals are inclined to engage in excessive avoidance to reduce uncertainties or increase the perception of control [31], which can lead to the overestimation of social exclusion. On the other hand, those individuals who perceive being socially excluded may have negative emotions. In particular, when confronting rejection, adolescents experience stronger negative emotions than adults [32]; thus, they are more sensitive to social relations and more worried about being excluded [33]. In this sense, it is possible that social exclusion is another essential mediator of the negative emotions produced by adolescents in response to the uncertainty created by the COVID-19 outbreak.

### 1.3. Perceived Efficacy as a Moderator

It is speculated that perceived efficacy may significantly moderate the relationship between IU and negative emotions. Perceived efficacy is made up of two parts: response efficacy and self-efficacy. In the pandemic context, response efficacy is an individual’s beliefs as to whether protective measures effectively prevent the threat; self-efficacy is an individual’s belief in his or her ability to carry out the recommended response [12,34,35]. Bandura [36] proposed that people’s beliefs in their coping capabilities play a pivotal role in the self-regulation of emotional states. Perceived efficacy is closely related to psychological health [37] and is a crucial factor protecting individuals from depression and anxiety [38,39,40]. High efficacy can buffer the negative effect of risk perception. For example, a study showed that efficacy and risk perception jointly predict subsequent health behaviors (e.g., issue salience, information seeking, preventive behavior). When at high risk, those with high efficacy tend to seek relevant information and adopt precautionary behaviors [41]. Likewise, perceived efficacy is a crucial protective factor helping adolescents withstand negative emotions [42,43] and promotes positive actions. Research has suggested that both response efficacy and self-efficacy can protect adolescents from substance use [44]. However, there are few studies investigating the moderation effect of perceived efficacy on social exclusion. Moreover, researchers have discovered that perceived efficacy plays a positive role in social support and prosocial behavior [45,46]. According to one study, people with higher efficacy are more prone to adhere to COVID-19-related precautionary measures such as physical distancing [47]. Considering that adolescents are in a special period in which they are seeking social identity and are sensitive to interpersonal relationships and rejection, the moderation effect of perceived efficacy between COVID-19 IU and social exclusion may be difficult to achieve. Therefore, how perceived efficacy plays a role in regulating negative emotions in adolescents still requires further exploration.

### 1.4. The Present Study

**Figure 1:**
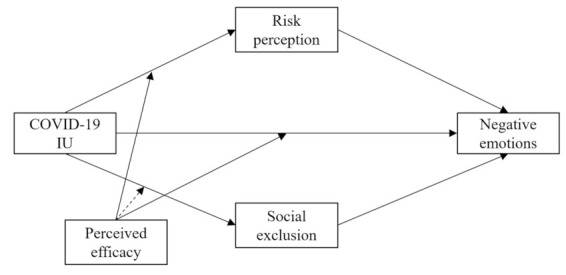
Hypothesized moderated mediation model.

To solve the problems mentioned above, this research establishes a moderated mediation model based on the integrative model of uncertainty tolerance (Figure 1). This model mainly proposes that COVID-19 IU has an indirect influence on adolescents’ negative emotions through the mediation of risk perception and social exclusion. The hypotheses are as follows: H1: COVID-19 IU is positively associated with negative emotions; H2a: COVID-19 IU positively predicts negative emotions through risk perception; H2b: COVID-19 IU positively predicts negative emotions through social exclusion; H3a: perceived efficacy negatively moderates the relationship between COVID-19 IU and risk perception; H3b: perceived efficacy might not moderate the relationship between COVID-19 IU and social exclusion; and H3c: perceived efficacy negatively moderates the relationship between COVID-19 IU and negative emotions. In summary, this study attempts to reveal the mechanism by which negative emotions are generated in adolescents during the COVID-19 pandemic, providing theoretical and evidence-based support for emotional regulation.

## 2. Materials and Methods

### 2.1. Participants

This study was approved by the Ethics Committee of the Institute of Psychology, Chinese Academy of Sciences. In the present study, participants were from a survey of psychological states conducted among members of the general public during the COVID-19 pandemic between 4 February and 6 February 2020 via the Tencent online survey platform. A total of 26,511 participants from 34 provinces or autonomous areas in China were recruited via WeChat to complete our questionnaire. In this study, we restricted the sample to participants aged 12–18 years because our study focused on the underlying mechanisms of the negative emotions generated in adolescents during the COVID-19 outbreak. Ultimately, 3037 adolescents were found eligible. Data collection was anonymous, and questionnaire information was kept confidential.

### 2.2. Measures

#### 2.2.1. Intolerance of COVID-19-Related Uncertainty

COVID-19 IU was assessed by two items that were adapted from a well-established questionnaire (i.e., the Intolerance of Uncertainty Scale, IUS [17]). They were “The uncertainty of the epidemic has severely impacted my life” and “It makes me uneasy, anxious, or stressed not having all the pandemic information I need.” Responses were rated on a 7-point Likert scale, ranging from 1 = strongly disagree to 7 = strongly agree (Cronbach’s *α* = 0.632).

#### 2.2.2. Negative Emotions

A considerable amount of research on both adults and adolescents indicates that anxiety, fear, and depression are key components of negative emotions [48,49,50]. Referring to the Positive and Negative Affect Schedule (PANAS) [51], we asked people to rate the intensity of three words that describe emotions and then give each negative emotion equal weight to calculate the total score. Additionally, previous research generally asked participants to report whether they had experienced each of the related symptoms during the prior one (e.g., Beck Depression Inventory and Self-Rating Anxiety Scale) or two (e.g., Patient Health Questionnaire-9) week; hence, we used the median. Correspondingly, negative emotions were measured by three questions: “In the last 10 days, what intensity of anxiety have you experienced?”, “In the last 10 days, what intensity of depression have you experienced?” and “In the last 10 days, what intensity of fear have you experienced?” Responses were rated on a 7-point Likert scale, ranging from 1 = very low to 7 = very high (Cronbach’s *α* = 0.864).

#### 2.2.3. Risk Perception

Risk perception includes two components based on the object being assessed. Universal risk perception is a general perception of fatality rates in the event of an outbreak in a specific area, while personal risk perception is one’s own specific probability of being infected with the virus or dying from the disease [52]. According to the previous literature related to the risk perception of infectious diseases, mortality and the likelihood of self-infection are two key indicators [53,54]. Hence, we designed two corresponding items, i.e., “In your opinion, how high is the fatality rate of COVID-19?” and “In your opinion, how likely are you to get infected with COVID-19?”. The questions were given equal weight and calculated to obtain a total score of risk perception. Responses were rated on a 7-point Likert scale, ranging from 1 = very low to 7 = very high (Cronbach’s *α* = 0.563).

#### 2.2.4. Social Exclusion

Social exclusion was measured by a single item, “COVID-19 patients have suffered social exclusion during this pandemic.” Responses were rated on a 7-point Likert scale, ranging from 1 = strongly disagree to 7 = strongly agree.

#### 2.2.5. Perceived Efficacy

We designed four items to reflect perceived efficacy reflecting the concepts of response efficacy and self-efficacy. These items were “I believe the pandemic will be fully controlled in the foreseeable future”, “I am confident that the pandemic will be overcome”, “To cope with the pandemic, I can discriminate between true information and rumors about COVID-19”, and “To combat the pandemic, I do not post or forward any messages that have not been officially confirmed about COVID-19”. These statements assess individuals’ beliefs as to whether preventive measures alleviate the pandemic and their beliefs in their ability to carry out the recommended response [34]. Responses were rated on a 7-point Likert scale, ranging from 1 = strongly disagree to 7 = strongly agree (Cronbach’s *α* = 0.787).

In summary, in this study, the items mentioned above were designed to reflect relevant variables in the context of the COVID-19 pandemic and exhibited good or acceptable reliability. In addition, all the participants completed a demographic questionnaire. The questionnaire included sex, age, education, concern time, and geographical location (whether in Hubei province at the epicenter of the outbreak).

### 2.3. Data Analysis

In this study, we first calculated descriptive statistics and performed Pearson’s correlation analysis with IBM SPSS statistics for Windows, Version 22.0 (IBM Corp, Armonk, NY, USA). Then, we used the SPSS macro PROCESS (Model 8) (http://www.afhyes.com) (access date 1 July 2020) suggested by Hayes [55] to test the moderated mediation model. All regression coefficients were tested by the bias-corrected percentile bootstrap method. Bootstrapping (5000 bootstrap samples) with 95% confidence intervals (CIs) was conducted to examine the significance of the mediation and moderation effects; 95% CIs that do not contain zero indicate that the effects are significant.

## 3. Results

### 3.1. Participants

**Figure 2:**
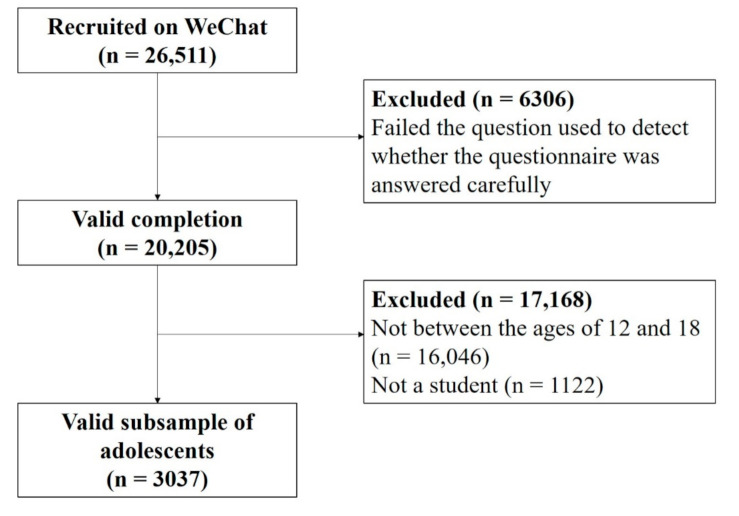
Flow diagram of the participant selection process.

In total, 26,511 people completed our survey via WeChat. In this study, the inclusion criteria were (1) correctly answering the questions used to test whether the questionnaire was carefully completed; (2) an age between 12 and 18 years; and (3) an occupation as a student. Figure 2 shows a flow diagram illustrating the numbers of individuals and reasons for exclusion at each inclusion stage. Finally, 3037 participants were included in the analyses. Because the online survey website set each question as “required to be answered” and the participants had to complete all questions before they could submit the questionnaire, our survey response rate was 100%. In previous survey analyses, in total, 166 articles reported mediation studies involving 189 independent samples; of these samples, the smallest sample size was 20, the largest sample size was 16,466, and only 9 studies used samples larger than 1500. In addition, the bias-corrected bootstrap had the highest power among six common methods used to detect the mediated effect. When using the bias-corrected bootstrap method, to obtain adequate power (0.8), low Type I error (α) and low Type II error (*β*), a sample size of 462 is needed according to empirical estimates of the sample size [56]. Thus, the sample size is adequate to test the moderated mediation model proposed in the present study.

The characterization of the study participants is presented below. The average age of the participants was 16.11 (SD = 1.68; range: 12–18), with 718 (23.64%) females and 2319 (76.36%) males. The respondents included 837 (27.56%) adolescents with a primary school education, 1915 (63.06%) high school students, 154 (5.07%) junior college students, and 131 (4.31%) undergraduate students. Among all participants, 548 (18.04%), 997 (32.83%), 822 (27.07%), 302 (9.94%), and 368 (12.12%) participants reported a daily concern time associated with COVID-19 information below 10 min, 11–30 min, 31–60 min, 61–120 min, and more than 120 min, respectively. In total, 111 (3.65%) adolescents were from Hubei province, i.e., the epicenter of the outbreak, and 2926 (96.35%) adolescents were from other provinces.

### 3.2. Preliminary Analyses

The descriptive statistics and correlations for all the variables are presented in Table 1. Specifically, COVID-19 IU was positively associated with risk perception (*r* = 0.30, *p* < 0.001), social exclusion (*r* = 0.40, *p* < 0.001), perceived efficacy (*r* = 0.31, *p* <0.001) and negative emotions (*r* = 0.38, *p* < 0.001). Both risk perception (*r* = 0.42, *p* < 0.001) and social exclusion (*r* = 0.35, *p* < 0.001) were positively correlated with negative emotions. Perceived efficacy was positively correlated with risk perception (r = 0.06, *p* < 0.001) and negatively correlated with negative emotions (*r* = −0.04, *p* < 0.02).

### 3.3. Testing the Proposed Model

Table 2 summarizes the main results of our moderated mediation model in five parts: Model 1, Model 2, Model 3, the conditional indirect effect analyses and the conditional direct effect analyses. Model 1 examined the effects of COVID-19 IU and perceived efficacy on risk perception, and Model 2 examined the effects of these variables on social exclusion. Model 3 was used to test the effects of COVID-19 IU, risk perception, social exclusion and perceived efficacy on negative emotions. Conditional indirect effect analyses were performed to test the effects of COVID-19 IU on negative emotions through the mediation of risk perception and social exclusion, respectively, at the mean of perceived efficacy as well as plus and minus one standard deviation from the mean of perceived efficacy. The conditional direct effect analyses tested the effects of COVID-19 IU on negative emotions at the mean of perceived efficacy as well as plus and minus one standard deviation from the mean of perceived efficacy.

After controlling for sex, age, education, concern time and province, the direct effect of COVID-19 IU on negative emotions was significant (B = 0.47, SE = 0.03, *p* < 0.001) (B, unstandardized coefficients; SE, standard error); moreover, COVID-19 IU was a positive predictor of risk perception (B = 0.30, SE = 0.02, *p* < 0.001) and risk perception was a positive predictor of negative emotions (B = 0.48, SE = 0.03, *p* < 0.001), suggesting that risk perception partially mediated the relationship between COVID-19 IU and negative emotions. Similarly, COVID-19 IU was a positive predictor of social exclusion (B = 0.29, SE = 0.01, *p* < 0.001) and social exclusion was a positive predictor of negative emotions (B = 0.35, SE = 0.04, *p* < 0.001), indicating that social exclusion played a partially mediating role in the relationship between COVID-19 IU and negative emotions. In addition, the interaction terms of COVID-19 IU and perceived efficacy showed significant effects on risk perception (B = −0.01, SE = 0.003, *p* = 0.002) and negative emotions (B = −0.01, SE = 0.01, *p =* 0.02). However, COVID-19 IU did not interact with perceived efficacy to predict social exclusion (B = −0.002, SE = 0.002, *p* = 0.23). Thus, these results showed that both the association between COVID-19 IU and risk perception and the association between COVID-19 IU and negative emotions were moderated by perceived efficacy, while perceived efficacy did not moderate the association between COVID-19 IU and social exclusion.

**Figure 3:**
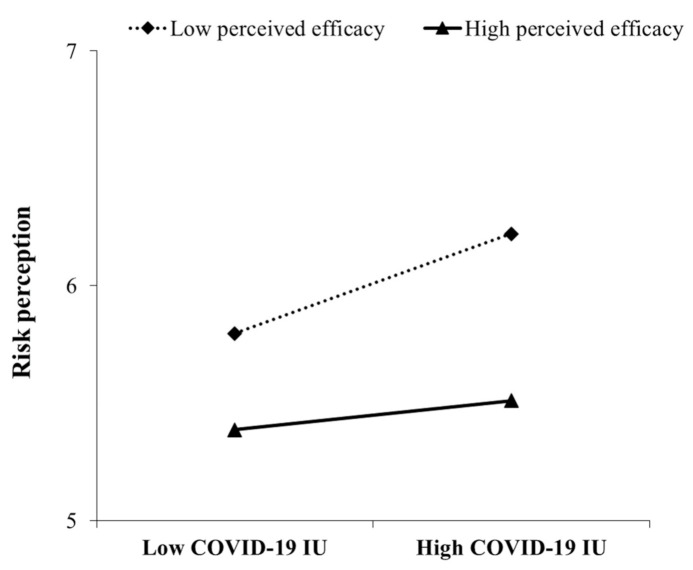
Perceived efficacy moderates the relationship between COVID-19 intolerance of uncertainty (IU) and risk perception.

**Figure 4:**
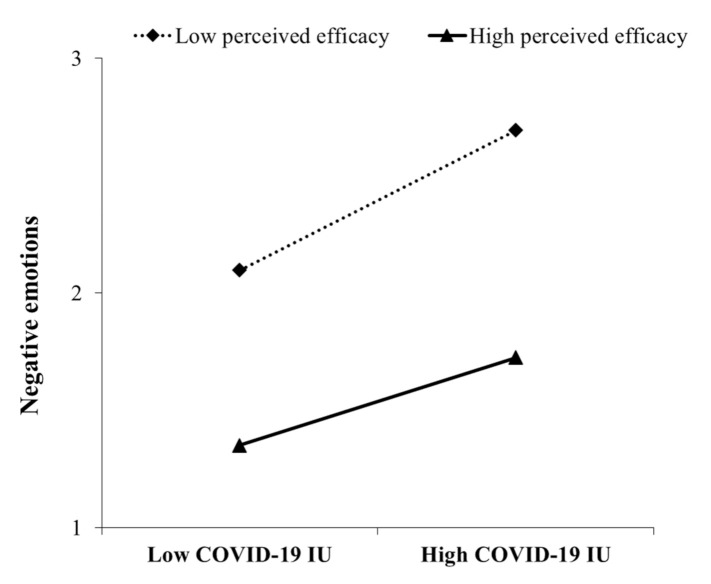
Perceived efficacy moderates the relationship between COVID-19 IU and negative emotions.

To further understand our moderated mediation model, two values of perceived efficacy, low perceived efficacy (i.e., one standard deviation below the mean) and high perceived efficacy (i.e., one standard deviation above the mean), were defined to test the moderating effects. As shown in Table 2, there was a significant indirect effect at every level of perceived efficacy, as all the CIs excluded 0. For individuals with high and low perceived efficacy, higher IU predicted higher risk perception. Nevertheless, the indirect effect on negative emotions through risk perception was larger for those with low perceived efficacy (indirect effect = 0.17, 95% CI = [0.14, 0.20]) than for those with high perceived efficacy (indirect effect = 0.12, 95% CI = [0.10, 0.15]) (see Figure 3). Likewise, the conditional direct effect analysis showed that perceived efficacy buffered the direct effect of COVID-19 IU on negative emotions, and compared to those with high perceived efficacy (direct effect = 0.42, 95% CI = [0.35, 0.48]), for those with low perceived efficacy, COVID-19 IU was a stronger predictor of negative emotions (direct effect = 0.52, 95% CI = [0.44, 0.61]) (see Figure 4). Overall, our findings show that perceived efficacy could alleviate the indirect effect of COVID-19 IU on negative emotions through the mediation of risk perception as well as the direct effect of COVID-19 IU on negative emotions.

## 4. Discussion

Few studies have explored negative emotions in adolescents during the COVID-19 pandemic. To fill this gap, this study focused on this special group and probed the predictive factors of and mechanisms underlying negative emotion among adolescents during the epidemic period. Our results show that COVID-19 IU was not only a positive and direct predictor of negative emotions in adolescents but was also positively associated with negative emotions via risk perception and social exclusion. Additionally, the direct relationship between COVID-19 IU and negative emotions proved to be moderated by perceived efficacy, as did the indirect path bridged by risk perception, which is consistent with our previous hypotheses. Namely, that higher efficacy lowered risk perception and negative emotions. However, regarding the indirect influence of COVID-19 IU on negative emotions through social exclusion, perceived efficacy exerted no significant moderation effect. In conclusion, this research tested a theoretical model of the relation between COVID-19 IU and negative emotions during the COVID-19 period and discovered the underlying mechanism of the effect of COVID-19 IU on negative emotions, providing evidence for the integrative model of uncertainty tolerance. From a practical perspective, since the variables involved are controllable, the results of this study are helpful for guiding the emotional self-regulation of adolescents and provide theoretical support for parents, schools, and governments seeking to intervene in the negative emotions of adolescents.

### 4.1. Associations between COVID-19 IU and Negative Emotions

As expected, our study found that COVID-19 IU was positively associated with negative emotions, which is consistent with previous findings [4,57,58]. Previous studies have also confirmed that IU is closely associated with generalized anxiety disorder [59,60]. People often experience frustration and sadness when facing uncertainties, and if their response is improper, they are more likely to experience excessive worry, anxiety and even depression. The COVID-19 pandemic, as a rare and uncertainty-laden event confronted by us all, has impacted every aspect of our lives; moreover, many governments have introduced unprecedentedly strict precautionary measures, which may be a source of serious stress. In particular, adolescence is characterized by a maladaptive shift in emotion regulation [61]. Adolescents may not be able to cope with the great uncertainty caused by the COVID-19 outbreak, resulting in more negative emotions such as anxiety, depression and fear.

### 4.2. The Mediating Role of Risk Perception

Our study shows that COVID-19 IU could predict negative emotions through the mediation of risk perception, which is consistent with the integrative model of uncertainty tolerance [11]. This study emphasizes that the overestimation of risk perception is associated with more negative emotions. On the one hand, individuals with high IU tend to be negative in assessing uncertainty [14], which in the pandemic context means overestimating the risk of being infected. People also sometimes pay close attention to pandemic-related information to decrease uncertainty, but consequently, they may perceive more risks [62]. On the other hand, excessive risk perception triggers excessive fear, anxiety and even depression. As COVID-19 has been considered infectious, destructive and recurring, adolescents become concerned about their health and that of their families when they find themselves unable to cope with the threat, leading to negative emotions.

### 4.3. The Mediating Role of Social Exclusion

Social exclusion was found to mediate the relationship between COVID-19 IU and negative emotions in adolescents. First, the present study demonstrates that high-IU individuals’ perception that patients are experiencing rejection may be related to their tendency to react negatively [14]. In addition, infectivity and public health measures (e.g., quarantine) might also strengthen the perception of social exclusion [63]. Second, the perception of social exclusion further predicts negative emotions in adolescents. Previous studies on adults have confirmed that social exclusion can increase individuals’ stress, threaten their fundamental needs (e.g., belonging and self-esteem), and lead to sadness and anger, causing them to become trapped in extreme misery [27]. It can be said that social exclusion has a considerable impact on emotions. Even when observing others suffering exclusion, people experience vicarious ostracism as if they were excluded and experience strong negative emotions. Previous studies have found that seeing others being excluded evokes negative feelings no less severe than those evoked by directly experiencing exclusion [64,65], which also occurs in adolescents [66]. Specifically, adolescents who are excluded may perceive a higher threat and experience greater declines in self-esteem than adults. As adolescents are at a stage when they are shifting from parent-centered relationships to peer-centered relationships, they long for acceptance and recognition from others. If these desires are not fulfilled, they develop negative outcomes, such as decreased self-control [67], depression, and anxiety [68,69]. During the special period of the COVID-19 crisis, adolescents have witnessed a pandemic unprecedented in scope and intensive defense measures, coupled with physical isolation and worry about social relationships, which results in substantial pressure [70]. Impaired social relationships in turn impair their self-regulation ability, leading to emotional and behavioral problems [71].

### 4.4. The Moderating Role of Perceived Efficacy

As the hypotheses state, the mediating effect of risk perception on the relation between COVID-19 IU and negative emotions was moderated by perceived efficacy, while the mediating effect of social exclusion was not. Previous studies have suggested that perceived efficacy, as a mental resource, helps individuals relieve pressure and respond with a more adaptive coping style [72,73,74]. Correspondingly, this study reconfirms the buffering effect of perceived efficacy on the associations among COVID-19 IU, risk perception and negative emotions. This effect is possible because individuals with high efficacy possess more mental resources to cope with threats. They are also more confident in the effectiveness of preventive measures and are willing to adopt infection-avoidance behaviors. Consequently, negative emotions caused by uncertainty are reduced. Nevertheless, perceived efficacy did not seem to buffer the negative effect of COVID-19 IU on social exclusion. This might be due to the existence of an automatic and sensitive system for detecting social exclusion in humans [75]. As small as the motion (e.g., averted eye gaze) might be, the sense of exclusion is still triggered [76]. This can also be explained by the specific conditions of adolescence. Since adolescents are in a critical period of social relationship development, they are vulnerable to social exclusion. Perceived efficacy’s protective effect is probably weakened by an overly strong perception of social exclusion and cannot buffer the negative influence of COVID-19 IU on social exclusion. In addition, studies have found that social exclusion weakens adolescents’ self-regulation ability [77], which makes it difficult for them to cope with negative emotions properly.

### 4.5. Limitations and Implications

The current study has several limitations. First, the sample is not balanced in terms of sex, which limits its representativeness. Therefore, we considered sex as a covariable in the data analysis to reduce its interference. Second, although the validity and reliability of the items used are acceptable, those of some variables were not sufficiently high. During the pandemic, isolation, lockdown, and other policies prevented offline investigations from being conducted. We should reduce the number of questions to the greatest extent possible to reduce time, increase enthusiasm, and ensure that the participants answer the questionnaire carefully. Third, although the test of negative emotion, such as anxiety, fear, and depression, in the current research is derived from the PANAS questionnaire, notably, these negative emotions are correlated but not exactly the same. In particular, the effect on behavior may even be the opposite. Therefore, future research should define the difference among negative emotions in adolescents more clearly and further investigate the influence of different negative emotions on epidemic prevention behavior. Fourth, considering the timeliness of COVID-19, only three experts evaluated the contents of the questionnaire while designing the questionnaire. Although the experts agreed that the current measurement content could better reflect the variables to be measured, additional content validity indexes need to be provided in the future. Fifth, to minimize the number of questionnaire items, only limited sociodemographic information was examined in the current study. However, such information can also influence adolescents’ negative emotions; thus, future research should examine more sociodemographic information, such as economic problems, family members affected or lost because of COVID-19, participation in social networks, beliefs about COVID, religious beliefs, etc.

Despite these limitations, to the best of our knowledge, the current study is the first to systematically investigate the mediating effect of risk perception and social exclusion on the relationship between COVID-19 IU and negative emotions and the moderating role of perceived efficacy in the same process. In addition, as a pioneer in the literature concerning adolescents during the pandemic, this research offers significant insights. First, it is indispensable to identify adolescents with high IU so that we can formulate measures in a timely manner to correct their cognitive bias. For example, cognitive behavioral therapy-intolerance of uncertainty (CBT-IU), which is an approach to treating IU that combines typical CBT components, including psychoeducation related to recognizing uncertainty, cognitive modifications of unrealistic positive illusions about seeking certainty, and exposure training for uncertainty, should be explored to enhance adolescents’ acceptance of the uncertainty of COVID-19 [78]. Next, considering that risk perception has ramifications for the effect of COVID-19 IU on negative emotions, it would be effective to help adolescents rationally understand risks and reduce the negative impact of risk perception by providing accurate knowledge of COVID-19 on the social networking platforms (e.g., WeChat, Weibo, and TikTok). Third, in the context of school closures, social distancing, and home quarantine [79,80], it is necessary to help adolescents correct their misconceptions that it is shameful to be socially excluded or have COVID-19 by strengthening ties with their peers, families and communities. Finally, improving adolescents’ response effectiveness and self-efficacy through intervention is also an effective way to buffer the adverse effects of COVID-19 IU. In particular, at the individual level, self-management and self-care strategies (e.g., problem solving, seeking information, acceptance, seeking support, and planning) could enhance their feelings of control and reduce their negative emotions in the face of the COVID-19 epidemic [81]. At the social level, the government should promote a positive anti-epidemic message, dispel rumors and provide adequate medical materials to enhance positive emotions among adolescents and, thus, improve self-efficacy in defeating the epidemic [34].

## 5. Conclusions

In summary, the findings of the current study extend our understanding of the integrative model of uncertainty tolerance to adolescents during the COVID-19 pandemic. The results of this study indicate that COVID-19 IU is positively associated with negative emotions in adolescents. These findings also provide additional information regarding the mechanisms underlying the relationship between COVID-19 IU and negative emotions among adolescents. Specifically, risk perception and social exclusion mediated the association between COVID-19 IU and negative emotions, indicating that higher levels of COVID-19 IU are more strongly correlated with risk perception and social exclusion and that, in turn, more negative emotions are experienced by adolescents. Furthermore, both the direct effect of COVID-19 IU and the indirect effect via risk perception on negative emotions were moderated by perceived efficacy, with a low level of perceived efficacy strengthening the two types of associations between COVID-19 IU and negative emotions.

## Figures and Tables

**Table 1:** Means, standard deviations, and correlations of all variables.

Variables	M	SD	1	2	3	4	5	6	7	8	9
1. Sex ^1^	-	-	1								
2. Age	16.11	1.68	0.14 **	1							
3. Education ^2^	-	-	0.06 **	0.34 **	1						
4. Concern time ^3^	-	-	0.04 *	−0.02	0.04 *	1					
5. Province ^4^	-	-	0.04 *	0.03	0.01	0.02	1				
6. COVID-19 IU	8.87	3.11	0.04 *	−0.06 **	0.01	0.05 **	−0.02	1			
7. Risk perception	7.18	3.04	−0.04	−0.16 **	0.01	0.06 **	−0.02	0.30 **	1		
8. Social exclusion	3.50	2.05	0.03	−0.13 **	0.06 **	−0.001	0.01	0.40 **	0.32 **	1	
9. Perceived efficacy	22.05	5.02	0.05 **	0.10 **	−0.03	0.10 **	0.02	0.31 **	0.06 **	−0.02	1
10. Negative emotions	10.08	5.12	−0.02	−0.14 **	0.04 *	0.07 **	−0.01	0.38 **	0.42 **	0.35 **	−0.04 *

Note. ^1^ Sex (0 = female, 1 = male). ^2^ Education (1 = primary education, 2 = high school, 3 = junior college, 4 = undergraduate). ^3^ Concern time (1 = “below 10 min”, 2 = “11–30 min”, 3 = “31–60 min”, 4 = “61–120 min”, 5 = “over 120 min”). ^4^ Province (0 = “other provinces”, 2 = “Hubei province”). * *p* < 0.05, ** *p* < 0.01.

**Table 2:** Conditional process analysis of the proposed moderated mediation model.

Model	B	SE	*t* Test	*p*-Value
**Model 1**				
Outcome: Risk perception				
Sex	−0.21	0.12	−1.66	0.10
Age	−0.29	0.03	−8.58	<0.001
Education	0.26	0.08	3.22	0.001
Concern time	0.12	0.04	2.74	0.006
Province	−0.18	0.28	−0.66	0.51
COVID-19 IU	0.30	0.02	16.06	<0.001
Perceived efficacy	−0.03	0.01	−2.15	0.03
COVID-19 IU × Perceived efficacy	−0.01	0.003	−3.10	0.002
**Model 2**				
Outcome: Social exclusion				
Sex	0.13	0.08	1.61	0.11
Age	−0.15	0.02	−6.74	<0.001
Education	0.26	0.05	5.07	<0.001
Concern time	−0.03	0.03	−1.05	0.30
Province	0.22	0.18	1.22	0.22
COVID-19 IU	0.29	0.01	24.05	<0.001
Perceived efficacy	−0.06	0.01	−7.67	<0.001
COVID-19 IU × Perceived efficacy	−0.002	0.002	−1.16	0.25
**Model 3**				
Outcome: Negative emotions				
Sex	−0.09	0.19	−0.50	0.62
Age	−0.18	0.05	−3.55	<0.001
Education	0.27	0.12	2.25	0.02
Concern time	0.22	0.06	3.50	<0.001
Province	−0.04	0.42	−0.10	0.92
COVID-19 IU	0.47	0.03	15.04	<0.001
Risk perception	0.48	0.03	17.09	<0.001
Social exclusion	0.35	0.04	8.04	<0.001
Perceived efficacy	−0.16	0.02	−8.67	<0.001
COVID-19 IU × Perceived efficacy	−0.01	0.005	−2.28	0.02
**Conditional indirect effect 1**	Effect	BootSE	BootLLCI	BootULCI
M − 1SD	0.17	0.02	0.14	0.20
M	0.14	0.01	0.12	0.17
M + 1SD	0.12	0.01	0.10	0.15
**Conditional indirect effect 2**	Effect	BootSE	BootLLCI	BootULCI
M − 1SD	0.11	0.02	0.07	0.14
M	0.10	0.01	0.07	0.13
M + 1SD	0.10	0.01	0.07	0.13
**Conditional direct effect**	Effect	SE	LLCI	ULCI
M − 1SD	0.52	0.04	0.44	0.61
M	0.47	0.03	0.41	0.53
M + 1SD	0.42	0.03	0.35	0.48

Note. Bootstrap sample size = 5000. LL = lower limit, CI = confidence interval, UL = upper limit; B = unstandardized coefficient. Conditional indirect effect 1 was COVID-19 IU → risk perception → negative emotions. Conditional indirect effect 2 was COVID-19 IU → social exclusion → negative emotions. Conditional direct effect was COVID-19 IU → negative emotions.

## Data Availability

The data presented in this study are available on request from the corresponding author.
